# A Photovoltaic-Integrated Broadband Photodetector Based on Vertically-Stacked Lateral-Aligned Nanowire Arrays

**DOI:** 10.3390/s25237308

**Published:** 2025-12-01

**Authors:** Ke Jin, Xin Yan, Yao Li, Xia Zhang

**Affiliations:** State Key Laboratory of Information Photonics and Optical Communications, Beijing University of Posts and Telecommunications, Beijing 100876, China; kjin@bupt.edu.cn (K.J.); liyao98@bupt.edu.cn (Y.L.); xzhang@bupt.edu.cn (X.Z.)

**Keywords:** photovoltaic-integrated photodetector, vertical stacking, Mie scattering, light trapping

## Abstract

A photovoltaic-integrated broadband photodetector based on vertically-stacked lateral-aligned III–V nanowire arrays is proposed and investigated. The staggered arrangement configuration drastically reduces the competition between solar cell and photodetector that is difficult to avoid in vertically-stacked planar structures, which enables broadband strong absorption. The lower GaAs nanowires (NWs) act as Mie scattering centers, which scatter the incident light passing through the gaps back to the upper layer, enhancing the absorption of InAs NWs over a wide wavelength range from the ultraviolet to the infrared. Meanwhile, the light trapping effect of the upper InAs nanowires improves the absorption of lower GaAs NWs. At a near-infrared wavelength of 1400 nm, the photovoltaic-integrated InAs nanowire photodetector exhibits a photocurrent density of 168.83 mA/cm^2^ and responsivity of 0.168 A/W, 90% and 93% higher than the single layer InAs nanowires. The conversion efficiency of the GaAs nanowire solar cell is also improved after integration. This work may pave the way for the development of self-powered miniaturized broadband photodetectors.

## 1. Introduction

Photodetectors have broad application prospects in fields such as communications [1,2,3,4], imaging [5,6], environmental monitoring [7,8], and biomedicine [9,10]. In recent years, with the rapid development of the Internet of Things (IoT) and low-altitude economy, miniaturization, lightweight, multifunctionality, low power consumption, and long-endurance have become important development trends for photodetectors and integrated systems [11,12,13]. Developing high-performance self-powered photodetectors and integrated systems that can operate independently, continuously, and efficiently without relying on external power supplies is emerging as an urgent need [14,15,16]. The most common approach to realize self-powered photodetectors is utilizing the photovoltaic effect at interfaces, and all of them use the built-in electric field in the junction to promote the separation of photogenerated carriers so as to realize light detection without bias voltage [17,18,19]. However, due to the limited strength of the built-in electric field, the photogenerated current and responsivity of these photovoltaic detectors at zero bias are generally lower than those of externally powered photodetectors [20]. High incident light power is required [21], severely limiting their high-sensitivity detection of weak optical signals.

In recent years, constructing self-powered integrated systems by harvesting different forms of energy from nature has become a research hotspot. Integrating energy harvesting units with photodetectors to drive photogenerated carrier separation can significantly enhance the device’s photocurrent and responsivity, achieving performance comparable to externally powered systems [22,23,24]. Among them, solar cells have outstanding advantages such as wide application scenarios, high stability, and long service life, and can provide a reliable guarantee of long-term efficient operation of equipment in complex environments. By integrating ZnO nanorod detectors and dye-sensitized solar cells side by side on a substrate, Young et al. showed a stable and repeatable transient response for the irradiation of 365 nm ultraviolet light [25], but this horizontally integrated structure still occupied a large area. Juan et al. formed a vertical stack structure of Ga_2_O_3_ thin film ultraviolet detector and crystalline silicon interdigitated back contact photovoltaic cell, which significantly reduced the device size, but the responsivity of the detector was only 9.8 × 10^−7^ A/W [26] due to the low voltage provided by solar cells. Hou et al. reported a self-powered ultraviolet detector with vertical stacking structure of GaInP/GaAs/Ge solar cells/ZnO nanowire arrays, and the device responsivity was only 3.39 × 10^−4^ A/W [27]. Obviously, vertical stacking of photodetectors and solar cells is a more compact integration mode. However, in order to avoid the competition between them in absorbing incident light, the reported detection wavelength is limited to the ultraviolet band, and the responsivity of photodetectors is generally low. Staggered arrangement of the upper and lower devices is a way to solve the above problems, and it has been reported that good results have been achieved [28].

This paper demonstrates a photovoltaic-integrated photodetector based on stacked lateral-aligned nanowire arrays (LNWAs). By staggered arrangement of the upper-layer InAs NW photodetector with the lower-layer GaAs NW solar cell, absorption competition for incident light is significantly reduced. By adjusting parameters such as the diameter and period of the upper and lower LNWA layers, the enhancement mechanism of the light trapping effect of the upper LNWA on the lower LNWA and the Mie scattering effect of the lower LNWA on the upper LNWA are investigated. Based on a set of optimized structural parameters, the stacked LNWA structure exhibits both enhanced photoresponsivity and photoelectric conversion efficiency, showing great potential in self-powered broadband photodetectors and integrated systems.

## 2. Materials and Methods

### 2.1. Device Structure

**Figure 1:**
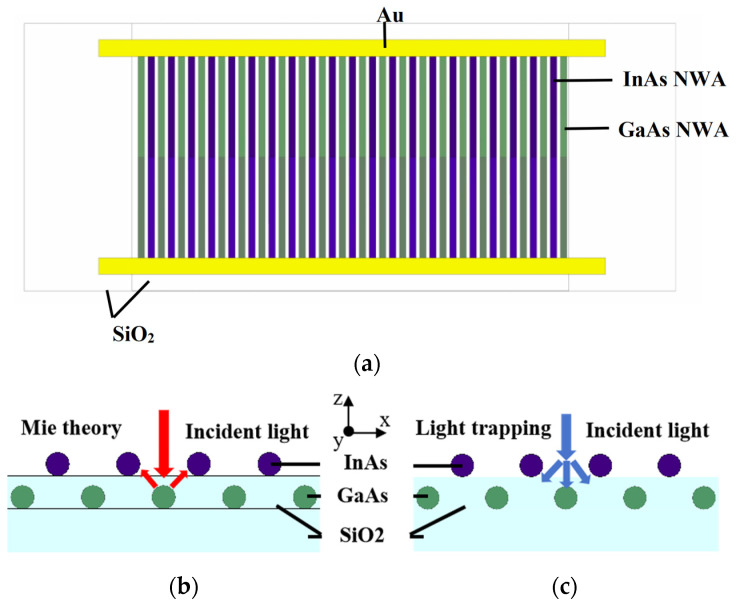
(**a**) Schematic diagram of the photovoltaic-integrated photodetectors. (**b**,**c**) Schematic of the Mie scattering and light trapping effect, respectively.

Figure 1a shows a schematic diagram of the integrated device based on stacked LNWAs. A SiO_2_ substrate with a thickness of 0.5 μm along the z-direction and a width of 6.5 μm along the y-direction is used at the bottom. A row of GaAs LNWA lying on the substrate serves as the solar cell, with a NW length of 5 μm consisting of a p-region length of 2.5 μm with a doping concentration of 2 × 10^19^ cm^−3^, and an n-region length of 2.5 μm with a doping concentration of 1 × 10^18^ cm^−3^. SiO_2_ is used as the cover layer encapsulating the GaAs LNWA. The InAs LNWA photodetector on the SiO_2_ cover layer has the same period as the lower-layer GaAs LNWA and is staggered arranged. The InAs LNWA has the same length, p–n junction structure, and doping level as the GaAs LNWA. The gap between two layers of LNWA is 0.1 μm. Both ends of the two layers of LNWA are connected in parallel using Au as electrodes and are connected outside the cover layer. This structure reduces the spatial absorption competition of incident light by staggered arrangement of two layers of LNWA. In addition, the two layers of LNWA reinforce each other through Mie scattering and light trapping, as shown in Figure 1b and c, respectively.

We use Lumerical FDTD Solutions and CHARGE Solutions to simulate and analyze the optical and electrical properties of InAs photodetectors and GaAs solar cells, respectively. And the version number of the software is 2020 R2. For optical simulations, periodic boundary conditions are used in the x-direction, while perfectly matched layers (PMLs) are used in the y- and z-directions for modeling. And The grid used for calculation in x-, y-, and z-directions are 5 nm, 10 nm, and 5 nm respectively. The wavelength-dependent complex refractive indices of GaAs, SiO_2_, and InAs can be obtained from [29,30,31]. An infinite extended plane wave is employed to simulate sunlight, with its parameters derived from the discrete AM 1.5 G solar spectrum. This light source covers a wavelength range of 0.3–1.7 μm and is incident along the -z direction. The results of transverse electric (TE) and transverse magnetic (TM) are superimposed to simulate the unpolarized characteristics of sunlight. The optical absorption per unit volume *P_abs_* and photogenerated carrier generation rate *g* can be calculated using the following formulas, respectively:

*P_abs_* = −0.5*ω*|*E*|^2^imag(*ε*) (1)

*g* = *P_abs_*/ћ*ω* (2)

where *ω* is the angular frequency of the incident light, *E* is the electric field strength, *ε* is the permittivity, and ћ is the reduced Planck constant.

In terms of electrical performance, the performance of the device before and after integration was compared by calculating the conversion efficiency *η* of the solar cells and the responsivity *R* of the photodetectors:

*η* = *P_MAX_*/*P_AM1.5G_* = (*FF* × *V_OC_* × *I_SC_*)/*P_AM1.5G_* (3)

*R* = (*I_light_* − *I_dark_*)/*P_in_A* (4)

where *P_MAX_* is the maximum output power, *P_AM1.5G_* is the solar power, *FF* is the fill factor, *V_OC_* is the open-circuit voltage, *I_SC_* is the short-circuit current, *I_light_* is the photocurrent, *I_dark_* is the dark current, *P_in_* is the incident light power density, and *A* is the effective area of the incident light.

### 2.2. Mie Theory

Mie theory is used in this paper to explain the absorption enhancement effect of the lower LNWA on the upper LNWA; there have been many studies on Mie theory of nanowires [32,33]. For the case that the incident light is vertically incident on the NW along the negative direction of z- axis, the scattering efficiencies *Q_sca_* for TE and TM polarizations can be expressed as [34]:

(5) $$Q_{\mathrm{sca},\mathrm{TE}}=2[{\left|a_{0}\right|}^{2}+2\sum_{i=1}^{\infty }{\left|a_{i}\right|}^{2}]$$

(6) $$Q_{\mathrm{sca},\mathrm{TM}}=2[{\left|b_{0}\right|}^{2}+2\sum_{i=1}^{\infty }{\left|b_{i}\right|}^{2}]$$

*a_i_* = [*mJ_i_*(*mx*)*J_i_*′(*x*) − *J_i_*′(*mx*)*J_i_*(*x*)]/[*mJ_i_*(*mx*)*H_i_*^(1)^′(*x*) − *J_i_*′(*mx*)*H_i_*^(1)^(*x*)] (7)

*b_i_* = [*J_i_*(*mx*)*J_i_*′(*x*) − *mJ_i_*′(*mx*)*J_i_*(*x*)]/[*J_i_*(*mx*)*H_i_*^(1)^′(*x*) − *mJ_i_*′(*mx*)*H_i_*^(1)^(*x*)] (8)

where *x* = *kr*, *k* = 2π/*λ*, *λ* is the wavelength of the incident light, *r* is the radius of the NW, *a_i_* and *b_i_* are Mie coefficients, *m* is complex refractive indexes, *J_i_* and *J_i_’* are the first Bessel functions and their derivatives, and *H_i_*^(1)^ and *H_i_*^(1)^’ are the first Hankel functions and their derivatives respectively.

## 3. Results and Discussion

**Figure 2:**
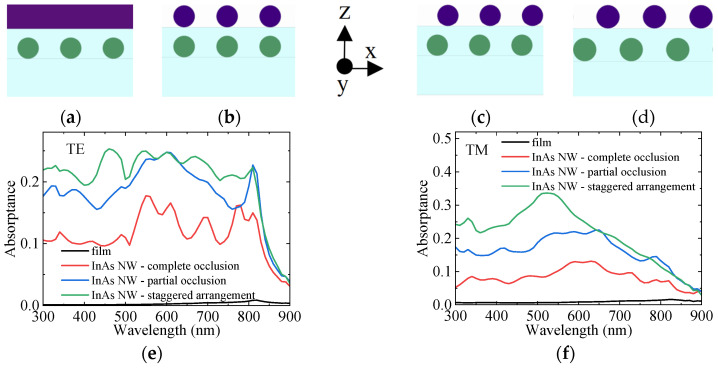
(**a**) GaAs NWs with an upper InAs thin film. (**b**) GaAs NWs with InAs NWs, where the two layers of NWs are completely vertically aligned. (**c**) GaAs NWs with InAs NWs, where the axes of the two layers of NWs are separated by 250 nm in the x-direction. (**d**) GaAs NWs with InAs NWs, where the two layers of NWs are arranged in a staggered manner. (**e**,**f**) Absorption of GaAs NWs in the four structures for (**e**) TE polarization, (**f**) TM polarization.

In order to illustrate the importance of staggered arrangement, Figure 2a–d studies the absorption of the GaAs nanowire array in four cases. The bottom layer is SiO_2_ with a thickness of 0.5 μm and a length of 6.5 μm along the axis of the nanowire as the substrate. Above the substrate is GaAs NWs with a length of 5 μm, a diameter of 500 nm, and a period of 1 μm. Above GaAs NWs is a SiO_2_ coating with a thickness of 100 nm and a length of 6.5 μm along the axis of the nanowire. In Figure 2a, the InAs thin film with a thickness of 500 nm and a length of 5 μm along the nano-axis is above the covering layer. In Figure 2b–d, it is InAs NWs with a diameter of 500 nm and a length of 5 μm. And in Figure 2b, InAs NWs completely blocks GaAs NWs. In Figure 2c, InAs NWs blocks half of GaAs NWs. In Figure 2d, InAs NWs and GaAs NWs are arranged in a staggered way, which is completely unobstructed. The absorption curves of GaAs NWs under TE and TM polarizations for the four cases are shown in Figure 2e,f, respectively. It can be seen that for both TE and TM polarizations, the absorption of GaAs NWs under the InAs film is the lowest, essentially approaching zero. When the InAs thin film is replaced by NWs, the absorption of GaAs is significantly enhanced. Moreover, as the InAs NWs move from directly above to the gap of GaAs NWs, the absorption increases accordingly. This is because reducing the shielding of the upper layer on the lower layer improves the absorption of the lower layer. When the upper layer is InAs thin film, the incident light can hardly reach the lower layer GaAs, resulting in almost zero absorption of GaAs. When the upper layer is InAs NWs, the gap of the upper layer array can let the incident light pass through, thus improving the absorption of the lower layer GaAs. Moreover, when the upper layers are nanowire arrays, the vertical alignment still has a great shielding effect on the lower GaAs, and only the more parts of the lower GaAs are in the gap between the upper layers, the higher the absorption will be. The above discussion demonstrates the necessity of using staggered arrangement, which helps to reduce the spatial absorption competition between the two layers of NWs, thereby enabling integrated devices with better performance.

### 3.1. Influence of GaAs LNWA on the Absorption of InAs LNWA

**Figure 3:**
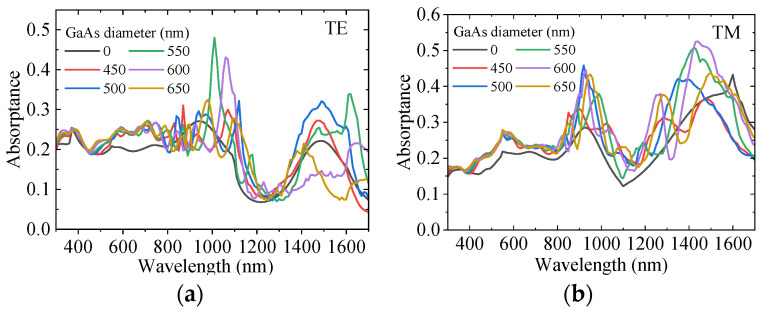
The absorption of InAs LNWA with different GaAs diameters for (**a**) TE and (**b**) TM polarized light, respectively.

**Figure 4:**
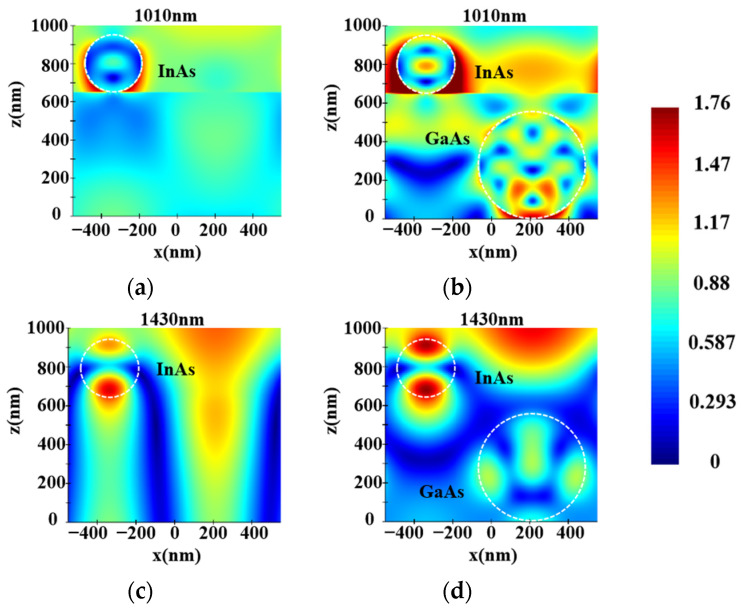
Electric field distributions of InAs LNWA in single and double-layer structures with a period of 1100 nm in the x–y plane. (**a**,**b**) TE polarization, incident wavelength at 1010 nm. (**c**,**d**) TM polarization, incident wavelength at 1430 nm.

According to Equations (5)–(8), the Mie scattering efficiency of NWs mainly depends on the incident light wavelength and NW diameter. This part investigates the influence of different GaAs LNWA diameters in the lower layer on the absorption of InAs LNWA in the upper layer when the incident wavelength is in the range of 0.3–1.7 μm. Due to the anisotropy of lateral NWs, the absorption characteristics for TE and TM incident polarizations are analyzed separately. We fix the diameter of the upper InAs LNWA at 300 nm and the period of both layers at 1100 nm. The absorption of InAs LNWA for TE and TM polarization varies with the diameter of GaAs LNWA, as shown in Figure 3a,b, respectively. It can be seen that GaAs with different diameters can enhance the absorption at most wavelengths before the wavelength of 1300 nm. However, in the 1300–1700 nm band, the enhancement effect cannot be achieved if the diameter is too large or too small. Moreover, for the same absorption curve, multiple absorption peaks appear across the entire spectrum, which all exhibit a redshift as the GaAs diameter increases. For example, when the GaAs diameter is 550 nm, strong absorption enhancements occur at wavelengths of 1010 nm, 1180 nm, and 1620 nm. However, when the GaAs diameter is increased to 600 nm, they occur at 1060 nm, 1230 nm, and 1650 nm. This is because the scattering efficiency of Mie theory will form multiple maxima in the whole wavelength range under a certain NW diameter. This phenomenon makes the backward scattering of GaAs at the corresponding wavelength stronger, thus increasing the propagation path length of incident light in InAs, thus increasing the absorption of InAs. In addition, the maximum scattering efficiency will appear as red shift with the increase of NW diameter. Correspondingly, the absorption enhancement peak of InAs will also be red-shifted with the increase of GaAs diameter [33]. Figure 4a–d show the electric field distributions in the x–y plane for TE and TM polarizations. As can be seen from Figure 4a,b, for a TE polarization incident wavelength of 1010 nm, the presence of the GaAs LNWA significantly enhances the electric field around the InAs LNWA, and the enhancement is mainly concentrated at the lower side of the InAs NW. Figure 4c,d show the electric field comparison for TM polarization, corresponding to the black and green lines at the 1430 nm wavelength in Figure 3b. Similarly, significant electric field enhancement is observed in Figure 4d.

**Figure 5:**
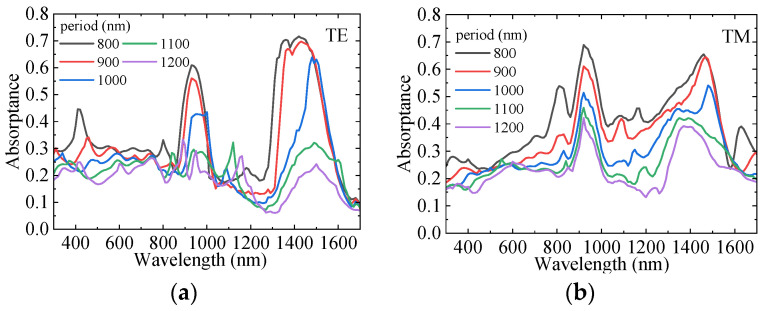
Dependence of the absorption of InAs LNWA on period for (**a**) TE polarization and (**b**) TM polarization.

To facilitate the subsequent optimization of integrated device parameters, the influence of period variation on the absorption of the upper-layer InAs LNWA are further studied. The InAs diameter is fixed at 300 nm, the GaAs diameter is set to 500 nm, and the period of the upper and lower LNWAs is scanned from 800 to 1200 nm. It can be seen from Figure 5 that, for both TE and TM polarizations, the absorption of InAs continuously decreases as the period increases. This is attributed to the reduced filling rate of the LNWA caused by the increased period, which leads to less light–matter interaction and weaker light-trapping capability, resulting in reduced absorption.

### 3.2. Influence of InAs LNWA on the Absorption of GaAs LNWA

**Figure 6:**
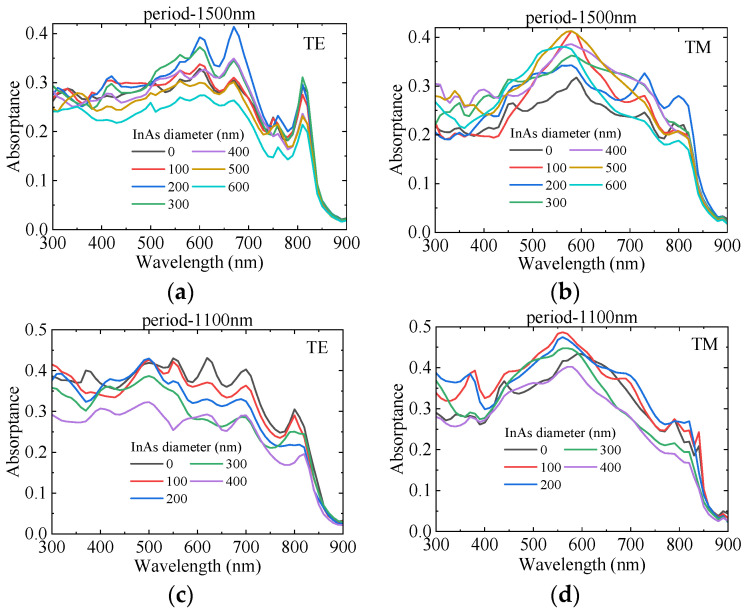
The absorption of GaAs LNWA with different InAs diameters and period for (**a**,**c**) TE and (**b**,**d**) TM polarized light, respectively.

**Figure 7:**
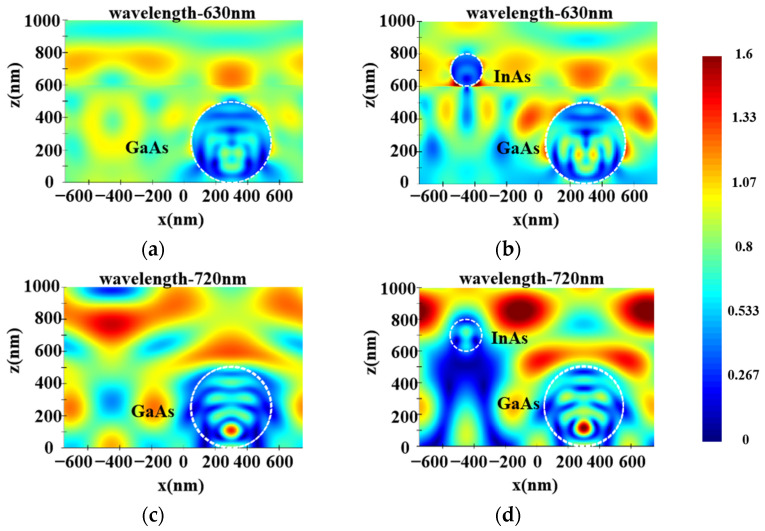
Electric field distributions of GaAs LNWA in single and double-layer structures with a period of 1500 nm in the x–y plane. (**a**,**b**) TE polarization, incident wavelength at 630 nm. (**c**,**d**) TM polarization, incident wavelength at 720 nm.

Figure 6 illustrates the effect of different InAs diameters on GaAs LNWA absorption. The GaAs diameter is fixed at 500 nm, with periods of 1100 nm and 1500 nm. At a period of 1100 nm, for TE polarization, GaAs absorption decreases with increasing InAs diameter. While for TM polarization, absorption first rises and then falls. At a period of 1500 nm, for both TE and TM polarizations, absorption first increases and then decreases with increasing InAs diameter. Meanwhile, as shown in Figure 6b–d, a broad-spectrum enhancement is observed rather than new absorption peaks. These phenomena indicate that the absorption enhancement of GaAs LNWA is not due to scattering but is caused by the light-trapping effect of the upper InAs LNWA. To further clarify the absorption enhancement mechanism of GaAs LNWA, electric field distribution are provided in Figure 7a–d. From these diagrams, it is evident that the introduction of InAs LNWA focuses the light wave around the GaAs LNWA, increasing the electric field and thus resulting in a higher light absorption.

**Figure 8:**
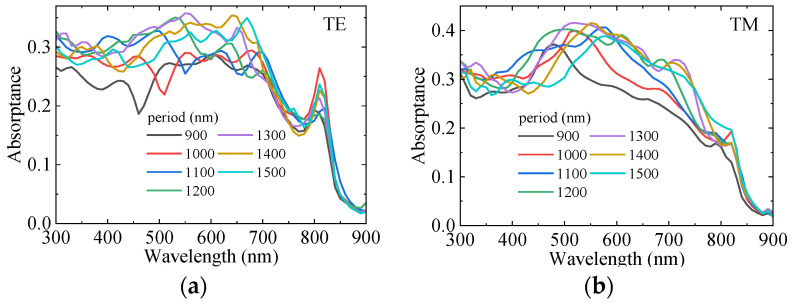
Dependence of the absorption of GaAs LNWA on period for (**a**) TE polarization and (**b**) TM polarization.

Figure 8 shows the variation of absorption of GaAs LNWA with period. The InAs LNWA diameter is set to 400 nm and GaAs LNWA diameter is set to 500 nm. From Figure 8a,b, it can be observed that with the increase of the period, the absorption of TE and TM polarizations exhibits a redshift. Moreover, the absorption peak of GaAs LNWA increases continuously with the increase of period, regardless of TE or TM polarization. However, when the period continues to increase (longer than 1300 nm at this parameter), the absorption peak decreases with the increase of the period. This indicates the competitive interaction between the light shielding and light trapping effect of the upper-layer InAs LNWA, which jointly affects the light absorption of the lower-layer GaAs LNWA. Reasonable design of structural parameters can minimize the reduction in absorption caused by shielding and leverage the light trapping effect to enhance the absorption.

### 3.3. Photoelectric Joint Simulation of the Integrated Device

Section 3.1 and Section 3.2 provide guidance for the design of the integrated device structure. For the period of double-layer LNWA, when the period is small, the absorption of InAs LNWA is high, but the absorption of GaAs LNWA will be significantly reduced due to serious shielding. For the diameter setting of InAs, an excessively large diameter will cause shielding of the lower layer, while a small diameter makes it difficult to form absorption enhancement for GaAs due to weak light trapping effects. For the diameter setting of GaAs, due to the influence of Mie scattering, there exists an optimal diameter that enhances the absorption of InAs. After analysis and design, the final parameters of the integrated device are set as InAs diameter of 300 nm, GaAs diameter of 500 nm, and a period of 1300 nm.

**Figure 9:**
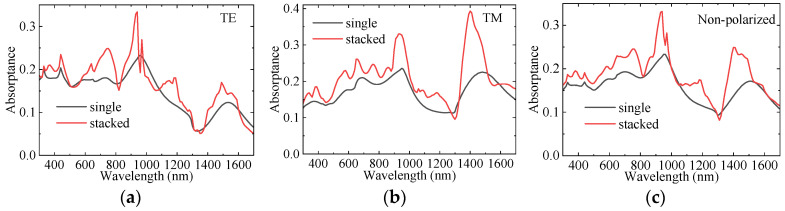
Comparison of InAs LNWA absorption before and after integration for (**a**) TE polarized light, (**b**) TM polarized light, and (**c**) Non-polarized light, respectively.

**Figure 10:**
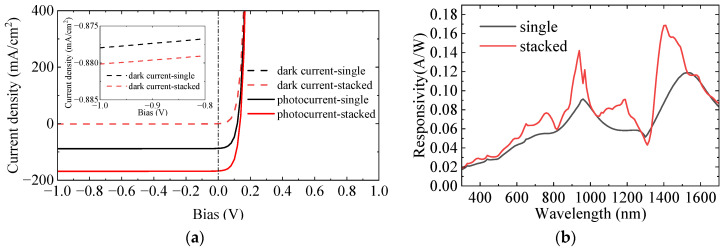
(**a**) I–V curve of InAs LNWA before and after integration at 1400 nm wavelength. (**b**) Responsivity of InAs LNWA before and after integration (at −0.5 V).

Figure 9a–c shows the absorption comparison of InAs LNWA before and after integration for non-polarized, TE, and TM polarizations. It can be seen that the integration of stacked NWAs achieves almost full-band absorption enhancement of InAs LNWA. As shown in Figure 9a, the absorption at 940 nm wavelength for TE polarization increases by 0.104, achieving a 46% improvement. For TM polarization, the absorption at 1400 nm wavelength increases by 0.191, with an improvement rate as high as 95%, as shown in Figure 9b. For non-polarized polarization, the absorption at 1400 nm is increased by 0.112, and the increase rate is 81%, as shown in Figure 9c. However, the absorption after integration is not improved at all wavelengths. For example, at the wavelength of 1300 nm, the absorption of TE, TM, and non-polarized all decreased in different degrees. This is because the introduction of GaAs NWs changes the effective refractive index of the whole device, which leads to the decrease of InAs absorption. To further evaluate the photodetection performance of InAs LNWA, Figure 10a,b shows the I–V curves and responsivity before and after integration, respectively. It can be seen that the dark current density of InAs before and after integration is basically the same. The photocurrent density increased from 88.73 mA/cm^2^ to 168.83 mA/cm^2^ at the wavelength of 1400 nm, an increase of about 90%. The responsivity is correspondingly increased from 0.087 A/W to 0.168 A/W, and the improvement rate reaches 93%.

**Figure 11:**
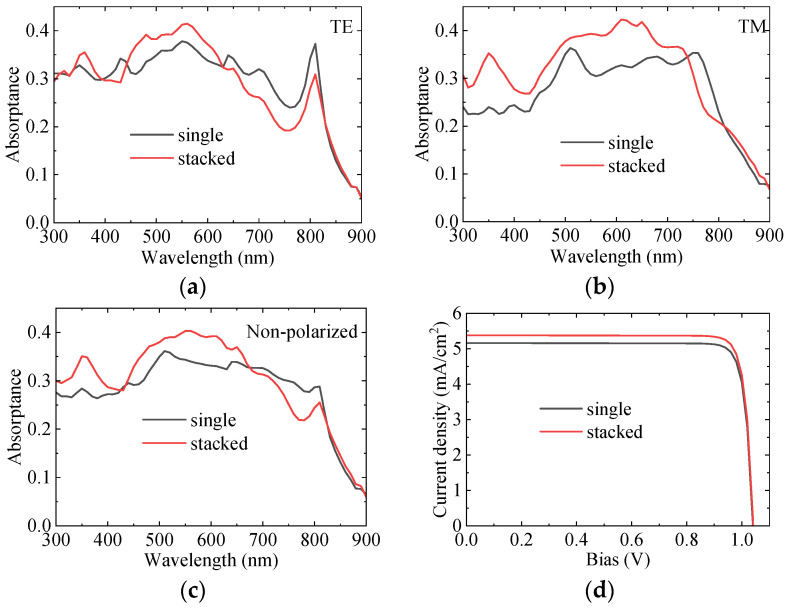
(**a**–**c**) Comparison of GaAs LNWA absorption before and after integration for (**a**) Non-polarized light, (**b**) TE polarized light, and (**c**) TM polarized light, respectively. (**d**) I–V curve of GaAs LNWA before and after integration.

Figure 11 shows the absorption and I–V curves of GaAs LNWA before and after integration. For TE polarization, before the wavelength of 630 nm, the absorption of the integrated device has been improved, as shown in Figure 11a. For TM polarization, this improved range is increased to 740 nm wavelength, as shown in Figure 11b. For the non-polarized case, the absorption is enhanced before the wavelength of 670 nm, as shown in Figure 11c. If the period of LNWA array is increased, the overall enhancement of GaAs at the wavelength of 300–900 nm can be realized, but the absorption of InAs will be sacrificed. As shown in Figure 11d, although the absorption around the wavelength of 750 nm has decreased, the overall short-circuit current density has increased from 5.16 mA/cm^2^ to 5.38 mA/cm^2^, and the conversion efficiency has therefore increased from 4.73% to 4.94%.

## 4. Conclusions

In summary, a InAs LNWA photodetector and GaAs LNWA solar cell are vertically stacked to form a photovoltaic-integrated self-powered photodetector. The staggered arrangement reduces the competition between the solar cell and photodetector, enabling broadband absorption ranging from ultraviolet to near-infrared. Moreover, the absorption of upper InAs LNWA and lower GaAs LNWA is enhanced due to the Mie scattering and light trapping effect, respectively. With optimized structural parameters, the stacked structure exhibits both enhanced photoresponsivity and photoelectric conversion efficiency, showing great potential in self-powered photodetectors. The stacked LNWA structure is expected to be extended to other self-powered devices such as sensors, light emitting diodes, and optoelectronic integrated devices.

The following is the prospect of the experiment. High-quality and flat SiO_2_ surface can be realized by plasma-assisted atomic layer deposition (ALD) [35], so as to be used as the substrate of LNWAs. An axial p–n junction NWs can be fabricated via molecular beam epitaxy (MBE) or metal-organic chemical vapor deposition (MOCVD). The as-grown NWs can be transferred onto the SiO_2_ substrate by isopropanol-assisted sonication, followed by precise alignment through micromanipulation [36]. These techniques can be used as reference schemes for subsequent experiments.

## Data Availability

The data presented in this study are available upon request from the corresponding author.
